# p53-Mediated Oxidative Stress Enhances Indirubin-3′-Monoxime-Induced Apoptosis in HCT116 Colon Cancer Cells by Upregulating Death Receptor 5 and TNF-Related Apoptosis-Inducing Ligand Expression

**DOI:** 10.3390/antiox8100423

**Published:** 2019-09-22

**Authors:** Matharage Gayani Dilshara, Ilandarage Menu Neelaka Molagoda, Rajapaksha Gedara Prasad Tharanga Jayasooriya, Yung Hyun Choi, Cheol Park, Kyoung Tae Lee, Seungheon Lee, Gi-Young Kim

**Affiliations:** 1 Department of Marine Life Science, Jeju National University, Jeju 63243, Korea; dilsharagm@gmail.com (M.G.D.);; 2 Department of Food Technology, Faculty of Technology, Rajarata University of Sri Lanka, Mihintale 50300, Sri Lanka; prasadrgtj@gmail.com; 3 Department of Biochemistry, College of Oriental Medicine, Dong-Eui University, Busan 47227, Korea; choiyh@deu.ac.kr; 4 Department of Molecular Biology, College of Natural Sciences and Human Ecology, Dongeui University, Busan 47340, Korea; 5 Forest Biomaterials Research Center, National Institute of Forest Science, Jinju 52817, Korea; leekt99@korea.kr

**Keywords:** indirubin-3′-monoxime, p53, death receptor 5, TNF-related apoptosis-inducing ligand, transcription factor C/EBP homologous protein

## Abstract

Indirubin-3′-monoxime (I3M) exhibits anti-proliferative activity in various cancer cells; however, its anti-cancer mechanism remains incompletely elucidated. This study revealed that I3M promotes the expression of death receptor 5 (DR5) and tumor necrosis factor (TNF)-related apoptosis-inducing ligand (TRAIL) in HCT116 p53^+/+^ cells, resulting in caspase-mediated apoptosis. However, this study demonstrated that HCT116 p53^−/−^ cells were insensitive to I3M-mediated apoptosis, indicating that I3M-induced apoptosis depends on the p53 status of HCT116 cells. Additionally, in HCT116 p53^-/-^ cells, I3M significantly increased Ras expression, while in HCT116 p53^+/+^ cells, it reduced Ras expression. Furthermore, I3M remarkably increased the production of reactive oxygen species (ROS), which were reduced in transient *p53* knockdown, indicating that I3M-mediated apoptosis was promoted by p53-mediated ROS production. Our results also showed that I3M enhanced transcription factor C/EBP homologous protein (CHOP) expression, resulted in endoplasmic reticulum (ER) stress-mediated DR5 expression, which was upregulated by ROS production in HCT116 p53^+/+^ cells. Moreover, co-treatment with I3M and TRAIL enhanced DR5 expression, thereby triggering TRAIL-induced apoptosis of HCT116 p53^+/+^ cells, which was interfered by a DR5-specific blocking chimeric antibody. In summary, I3M potently enhances TRAIL-induced apoptosis by upregulating DR5 expression via p53-mediated ROS production in HCT116 p53^+/+^ cells. However, HCT116 p53^−/−^ cells were less sensitive to I3M-mediated apoptosis, suggesting that I3M could be a promising anti-cancer candidate against TRAIL-resistant p53^+/+^ cancer cells. Additionally, this study also revealed that I3M sensitizes colorectal cancer cells such as HT29 and SW480 to TRAIL-mediated apoptosis.

## 1. Introduction

Apoptosis is the process of programmed cell death characterized by distinct morphological and genetic alterations, including cell shrinkage, chromatin condensation, and DNA fragmentation [1]. For several decades, the tumor suppressor p53, has been considered as a main tumor-removing molecule, exerting its effects via cell cycle arrest and apoptosis. However, approximately 55% of all human cancers carry a mutated or silenced *p53* gene [2]. Therefore, p53 upregulation at cellular levels has been recognized as a promising strategy for cancer treatment, and is currently being evaluated in preclinical and clinical trials [3]. Cellular p53 levels induce the production of reactive oxygen species (ROS), which in turn can also provide a positive feedback to cellular p53 production [4]. Particularly, hyperphysiological p53 levels trigger pro-oxidant enzymes and increase ROS generation, inducing ROS-mediated apoptosis [5]. Additionally, a recent study showed that p53 activation sensitizes tumor necrosis factor-related apoptosis-inducing ligand (TRAIL)-mediated apoptosis by upregulating the expression of C/EBP-homologous protein (CHOP)-mediated death receptor (DR) 4 and 5, which increases pro-apoptotic protein expression and ROS generation [6]. Therefore, targeting p53 stimulation can be a promising strategy for cancer treatment.

Indirubin is an active ingredient of Dang Gui Long Hui Wan, a mixture of herbal medicines commonly used in traditional Chinese medicine to treat chronic myelocytic leukemia (CML) [7]. For several decades, a number of indirubin derivatives and analogues have been synthesized and developed to boost its promising anti-cancer activity, and its commercially available analogue indirubin-3′-monoxime (I3M), is reported to strongly inhibit the growth of various human cancer cells, including human non-small cell lung [8], human pancreatic [9], and renal [10] cancer cells. Additionally, glycogen synthase kinase-3β (GSK-3β) and cyclin-dependent kinase (CDK) are known as molecular targets of I3M, which exert anti-mitotic properties by inducing endoreduplication following prophase arrest [11]. However, the possibility of a direct association between p53 and I3M-mediated anti-cancer activities remains unclear.

This study revealed that I3M enhanced p53-mediated oxidative stress, which triggered TRAIL-mediated apoptosis by activating CHOP-mediated DR5 expression in wild type HCT116 human colon cancer cells. Additionally, the current study revealed that I3M sensitizes colorectal cancer cells such as HT29 and SW480 to TRAIL-mediated apoptosis. The results of this study also suggest the possibility of co-treatment of human colon cancers with I3M and TRAIL to treat human colon cancers dependent on the p53 status.

## 2. Materials and Methods

### 2.1. Materials and Reagents

I3M was purchased from Tocris Bioscience (Bristol, UK). The followings were purchased from Sigma Chemical Co. (St. Louis, MO, USA): 3-(4,5-Dimethyl-2-thiazolyl)-2,5-diphenyl-2H-tetrazolium bromide (MTT), *N*-acetylcysteine (NAC), 4′6-diamidine-2′phenylindole dihydrochloride (DAPI), 2′7′-dichlorofluorescin diacetate (DCFDA), and z-VAD-fmk. Anti-human antibodies against Bid (SC-373939), Bax (SC-493), caspase-3 (SC-7148), Ras (SC-32), p53 (SC-1311), DR5 (SC-53689), TRAIL (SC-8410), CHOP (SC-7351), and β-actin (SC-69879) were purchased from Santa Cruz Biotechnology (Santa Cruz, CA, USA). Peroxidase-labeled donkey anti-rabbit and sheep anti-mouse immunoglobulin, and recombinant human TRAIL/Apo2 ligand (the nontagged 19-kDa protein, amino acids 114–281) were purchased from KOMA Biotechnology (Seoul, Republic of Korea). DR5/Fc chimera protein was purchased from R&D Systems (Minneapolis, MN, USA). Roswell Park Memorial Institute (RPMI) 1640 medium, antibiotic mixture, and fetal bovine serum (FBS) were obtained from WelGENE Inc. (Daegu, Korea).

### 2.2. Cell Culture

p53-Null (p53^−/−^) and wild-type (p53^+/+^) human HCT116, HT29, and SW480 colon carcinoma cells were obtained from the American Type Culture Collection (Manassas, VA, USA) and maintained in RPMI 1640 medium supplemented with 10% heat-inactivated FBS and 1% penicillin-streptomycin in 5% CO_2_ at 37 °C. Periodically, the cells were tested for mycoplasma contamination using a MycoAlert Mycoplasma Detection Kit (Lonza, Rockland, ME, USA).

### 2.3. Cell Viability Assay

Relative cell viability was measured using 3-(4,5-Dimethyl-2-thiazolyl)-2,5-diphenyl-2H-tetrazolium bromide (MTT) assay based on the conversion of MTT to formazan crystals by mitochondrial dehydrogenases, which has been used as a surrogate for viability. HCT116 p53^−/−^ and p53^+/+^ colon cancer cells (1 × 10^5^ cells/mL) were pretreated for 24 h and then incubated with 0.5 mg/mL MTT for 45 min at 37 °C. The culture medium was removed and dimethyl sulfoxide was added to each well to dissolve formazan. The absorbance was measured at 540 nm using a microplate reader. The relative cell viability (%) was expressed as a percentage relative to the untreated control cells.

### 2.4. Western Blotting Analysis

HCT116 cells were harvested by scraping from the wells and washed twice with cold phosphate buffered saline (PBS). Total cell extracts were prepared using a PRO-PREP^TM^ protein extraction kit (iNtRON Biotechnology, Seongnam-si, Gyeonggi-do, Korea). Lysates were centrifuged at 16,000 rpm at 4 °C for 30 min to obtain the supernatants. Protein concentration was determined by a Bio-Rad protein assay kit (Bio-Rad, Hercules, CA, USA), loaded on 10% SDS-PAGE, and electrophoretically transferred to a polyvinylidene difluoride membrane. The membrane was incubated with primary antibodies overnight at 4 °C, washed three times, and reincubated with peroxidase-labeled secondary antibody for 2 h. A chemiluminescence (ECL) western blot kit (Amersham, Arlington Heights, IL, USA) was used to detect specific proteins.

### 2.5. Flow Cytometry Analysis

HCT116 cells were treated with I3M, trypsinized, and washed in cold 1 × PBS. The cells were then incubated with MUSE^®^ annexin V and dead cell assay reagent (EMD Millipore, Billerica, MA) for 20 min and the early/late apoptotic cell population was measured by MUSE^®^ cell cycler (EMD Millipore, Billerica, MA, USA).

### 2.6. Reverse Transcription-Polymerase Chain Reaction (RT-PCR)

Total RNA was extracted using Easy-Blue reagent (iNtRON Biotechnology) according to the manufacture’s protocol. RNA was reverse-transcribed to cDNA using the One-Step RT-PCR Premix (iNtRON Biotechnology). Synthesized single stand cDNA was amplified by RT-PCR with the following primer pairs: *DR5* forward, 5′-AAG ACC CTT GTG CTC GTT GTC-3′, *DR5* reverse 5′-GAC ACA TTC GAT GTC ACT CCA-3′, *CHOP* forward 5′-CAA CTG CAG AGA TGG CAG CTG A-3′ and *CHOP* reverse 5′-CTG ATG CTC CCA ATT GTT CAT-3′, *glyceraldehyde-3-phosphate dehydrogenase* (*GAPDH*) forward 5′-GTC TTC ACC ACC ATG GAG-3′ and *GAPDH* reverse 5′-CCA CCC TGT TGC TGT AGC-3′. The reaction sequence consisted of 50 °C for 30 min, 94 °C for 2 min, and 94 °C for 29 cycles of 15 s each; 60 °C for 30 s; and 72 °C for 45 s with an extension at 72 °C for 10 min. PCR products were analyzed by electrophoresis on 1% agarose gel and expression levels of each molecule were normalized to *GAPDH* level within the same sample.

### 2.7. Transient Knockdown of p53 and CHOP

HCT116 cells were transfected with *p53* and *CHOP*-specific silencing RNA (sip53 and siCHOP) as well as control siRNA (siCON) (Santa Cruz Biotechnology) for 48 h. For each transfection, 20 nM siRNA duplex with the transfection reagent G-Fectin (Genolution Pharmaceuticals, Inc., Seoul, Korea) was added in 450 μL of growth medium and the entire mixture was added gently to the cells.

### 2.8. Fluorescence Microscopy

HCT116 cells were fixed with 3% formaldehyde for 15 min, followed by permeabilization with 0.5% Triton X-100 for 15 min. The cells were then blocked for 1 h with 2% bovine serum albumin and stained with rabbit monoclonal anti-human DR5 overnight at 4 °C. The cells were washed and then incubated with Alexa Fluor^®^ 488-conjugated IgG. Nuclei were counterstained with 4′6-diamidine-2′phenylindole dihydrochloride (DAPI). The cells were visualized using fluorescence microscopy. For the live cell imaging, HCT116 cells were cultured in 8 well chamber slides and after chemical treatment, cells were washed with PBS and stained with 10 μM 2′7′-dichlorofluorescin diacetate (DCFDA). Live imaging was performed with CELENA^®^ S digital imaging system (Logos biosystems; Anyang-si, Gyeonggi-do, Korea).

### 2.9. Statistical Analysis

All values are expressed as means ± the standard error of the mean (SEM). The data were derived from at least three independent experiments. Multiple group comparison was performed by the Student’s t-test and a one-way ANOVA test. The images were visualized with Chemi-Smart 2000 (Vilber Lourmat, Cedex, France). Images were captured using Chemi-Capt (Vilber Lourmat, Marne-la-Vallee, France) and transported into Adobe Photoshop (version 8.0). Values of *p* < 0.001 were considered to be statistically significant.

## 3. Results

### 3.1. I3M-Induced HCT116 Apoptosis Is Dependent on p53 Expression

**Figure 1:**
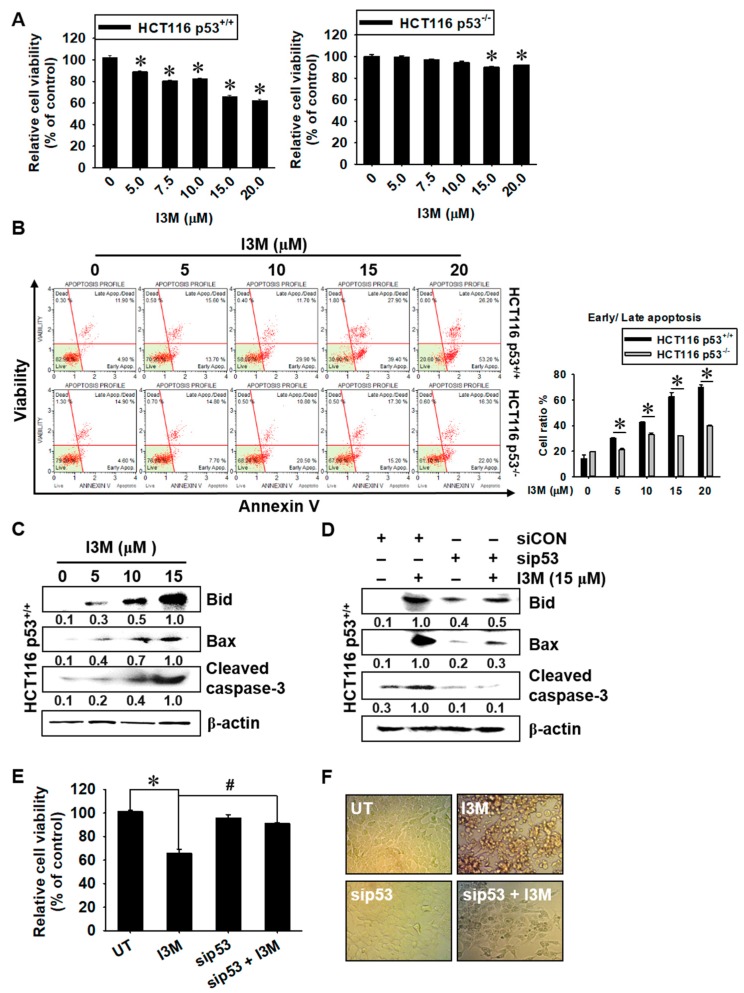
Effect of I3M on wild-type (p53^+/+^) and p53-null (p53^−/−^) human HCT116 colon cancer cell viability. HCT116 p53^+/+^ and HCT116 p53^−/−^ cells were treated with various concentrations of I3M (0-20 μM). (**A**) Cell viability was measured using the MTT assay, 24 h after I3M treatment. (**B**) In a parallel experiment, early and late apoptotic cell populations were measured using MUSE^®^ annexin V and dead cell assay kit by flow cytometry. The apoptotic percentages of each HCT116 p53^+/+^ and HCT116 p53^−/−^ cells are indicated in each panel (right) and total apoptotic cell percentages are shown in bar graph (left). (**C**) Protein lysates from HCT116 p53^+/+^ cells were prepared at 24 h, subjected to SDS-PAGE, and immunoblotted using specific antibodies against Bid, Bax, and cleaved caspase-3. (D-F) HCT116 p53^+/+^ cells were transiently transfected with *p53* siRNA for 48 h and then treated with 15 μM I3M for 24 h. (**D**) Western blotting was performed to determine Bid, Bax, and cleaved caspase-3. β-Actin was used as an internal control for western blotting. (**E**) Cell viability was measured using the MTT assay. Cell morphology was examined under a light microscope (**F**). The results are the average of three independent experiments and are presented as the mean ± SEM. *, *p* < 0.001 vs. the untreated control (UT) and #, *p* < 0.001 vs. the I3M-treated group.

To investigate whether I3M-induced apoptosis depends on p53 status, HCT116 p53^+/+^ and HCT116 p53^−/−^, the cells were treated with I3M for 24 h, and then relative cell viability using mitochondrial activity and annexin V staining were evaluated. I3M treatment decreased HCT116 p53^+/+^ cell viability in a dose-dependent manner compared with the untreated group (90.0 ± 0.9%, 80.5 ± 0.8%, 81.2 ± 0.7%, 66.1 ± 1.1%, and 62.5 ± 0.9% cell viability at 5, 7.5, 10, 15, and 20 µM I3M doses, respectively) (Figure 1, left). However, both I3M treatment at 15 and 20 μM slightly downregulated HCT116 p53^-/-^ cell viability at over 90% compared with the untreated group (Figure 1, right). Additionally, annexin V staining data showed that significant annexin V^+^ and dead cell marker^-^ (early apoptosis; lower-right) and annexin V^+^ and dead cell marker^+^ (late apoptosis; upper right) populations, which represent apoptosis, were present in I3M-treated HCT116 p53^+/+^ cells (30.1 ± 3.1%, 42.7 ± 0.6%, 62.8 ± 3.1%, 69.9 ± 1.9% at 5, 10, 15 and 20 µM I3M, respectively) (Figure 1B, top). However, a negligible population was present in I3M-treated HCT116 p53^−/−^ cells (Figure 1B, bottom), suggesting that I3M-induced apoptosis depends on the p53 status of HCT116 cells. Additionally, 15 μM I3M treatment significantly induced the expression of apoptotic proteins, including Bid, Bax, and cleaved caspase-3, in HCT116 p53^+/+^ cells (Figure 1C). In addition, to evaluate the association of p53 status with I3M-mediated apoptosis, HCT116 p53^+/+^ cells were transiently transfected with sip53, and then apoptotic protein expression, cell viability, and cellular morphology were investigated. Transient p53 knockdown significantly attenuated I3M-induced expression of Bid, Bax, and caspase-3 in HCT116 p53^+/+^ cells, indicating that p53 is the main I3M-mediated apoptosis inducer (Figure 1D). Additionally, I3M-mediated HCT116 p53^+/+^ cell viability downregulation was restored by up to 90% by transient p53 knockdown, compared with the untreated control group (Figure 1E). sip53 transfection also alleviated I3M-induced rounding and detaching of HCT116 p53^+/+^ cells (Figure 1F). Taken together, these data indicate that I3M-mediated apoptosis is dependent on the p53 status of HCT116 cells.

### 3.2. I3M Enhances p53 Expression in HCT116 p53^+/+^ Cells, Accompanied by Ras Downregulation

**Figure 2:**
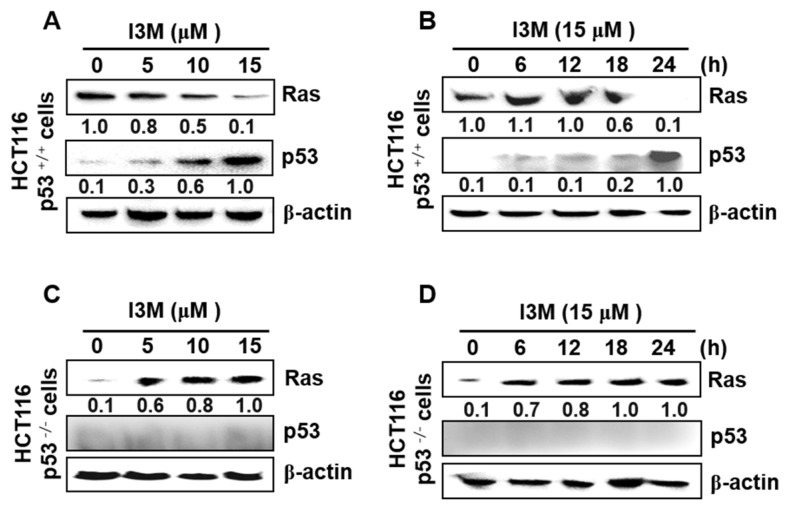
Effect of I3M on Ras and p53 expression in HCT116 p53^+/+^ and HCT116 p53^−/−^ cells. (**A**,**B**) HCT116 p53^+/+^ cells were treated with the indicated I3M concentrations (0–15 μM) for 24 h (**A**) and with 15 μM I3M for the indicated time (0–24 h) (**B**). Ras and p53 expression were then detected using western blotting. (**C**,**D**) HCT116 p53^−/−^ cells were treated with the indicated I3M concentrations (0–15 μM) for 24 h (**C**) and with 15 μM I3M for the indicated times (0–24 h) (**D**). Ras and p53 expression were detected using western blotting. β-Actin was used as a loading control. The results are the average of three independent experiments and representative relative density mean values are indicated.

Ries et al., showed that by inducing mouse double minute 2 homolog (Mdm2) in mouse embryo fibroblasts, oncogenic Ras degrades p53 [12], indicating that Ras activation decreases p53 expression, leading to cancer cell survival. Therefore, this study elucidated the expression of Ras and p53 in I3M-mediated apoptosis. I3M significantly downregulated Ras expression in a dose-dependent manner and increased p53 expression in HCT116 p53^+/+^ cells (Figure 2A). Additionally, 24 h after 15 μM I3M treatment, Ras expression in HCT116 p53^+/+^ cells was significantly downregulated, resulting in a remarkable increase in p53 levels (Figure 2B). To confirm the critical role of p53 in Ras expression, HCT116 p53^−/−^ cells were treated with I3M and Ras expression significantly increased in HCT116 p53^−/−^ cells, both in a time-dependent (Figure 2C) and a dose-dependent (Figure 2D) manner. These results indicate that I3M induces p53 expression and suppresses Ras expression, leading to cell death.

### 3.3. I3M-Induced DR5 Expression Depends on p53 Expression

**Figure 3:**
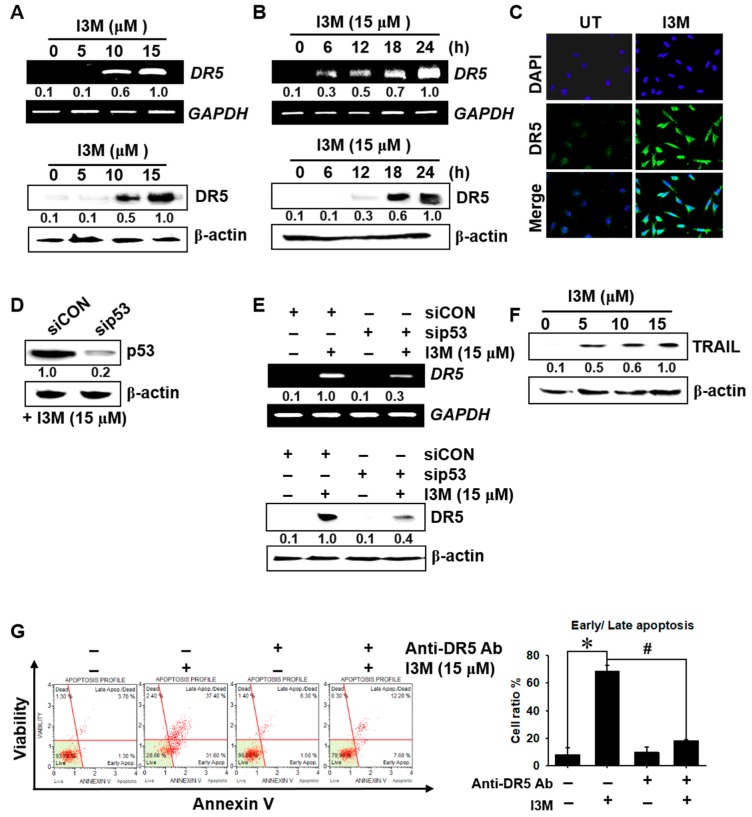
I3M upregulates DR5 expression in HCT116 p53^+/+^ cells via p53 activation. (**A**) HCT116 p53^+/+^ cells were seeded at 1 × 10^5^ cells/mL and incubated with the indicated I3M concentrations (0–15 μM). (**B**) The cells were incubated with I3M (15 μM) for the indicated time (0-24 h). RT-PCR and western blotting for DR5 were performed at 12 h and 24 h, respectively. (**C**) HCT116 p53^+/+^ cells treated with 15 μM I3M for 24 h. The cells were fixed, permeabilized, and stained with DR5 monoclonal antibody, which was detected using an anti-mouse secondary antibody conjugated with Alexa Fluor^®^ 488 and then stained with 4′6-diamidine-2′phenylindole dihydrochloride (DAPI) solution. The stained DR5 and nuclei were then observed under a fluorescent microscope (×400). (**D**,**E**) HCT116 p53^+/+^ cells were transiently transfected with *p53* siRNA (sip53) for 48 h (**D**) and then treated with 15 μM I3M for 24 h (**E**). RT-PCR and western blotting were performed to detect DR5 expression, glyceraldehyde-3-phosphate dehydrogenase (*GAPDH*) and β-actin used as internal controls for RT-PCR and western blotting, respectively. (**F**) Equal amounts of HCT116 p53^+/+^ cell lysates were separated using SDS-PAGE and tumor necrosis factor (TNF)-related apoptosis-inducing ligand (TRAIL) expression was determined using western blotting, and β-actin was used as a loading control. (**G**) DR5 blocking chimera antibody was pretreated 2 h before I3M treatment for 24 h. Early and late apoptotic cell populations were measured using MUSE^®^ flow cytometer. The results are the average of three independent experiments and representative relative density mean values are indicated. *, *p* < 0.001 vs. the untreated control (UT) and #, *p* < 0.001 vs. the I3M-treated group.

Given that p53 is known to directly transactivate DR5 expression by binding to an intronic sequence-specific DNA-binding site [13], which triggers TRAIL-mediated apoptosis, I3M involvement in p53-mediated DR5 expression and apoptosis was investigated. We found that >10 μM I3M significantly increased DR5 expression at both the transcriptional (top) and translational (bottom) levels (Figure 3A). Additionally, I3M (15 μM) treatment of HCT116 p53^+/+^ cells remarkably increased DR5 mRNA and protein levels at 18 h, reaching a maximum at 24 h (Figure 3B). Confocal microscopic analysis also confirmed that I3M significantly induced DR5 expression in HCT116 p53^+/+^ cells (Figure 3C). To further verify whether DR5 expression is regulated via p53, sip53 was transfected into HCT116 p53^+/+^ cells, and then western blotting was performed. This analysis showed that sip53 transfection remarkably depleted p53 expression compared with the siCON-treated group (Figure 3D). Transient *p53* knockdown significantly downregulated I3M-induced DR5 mRNA and protein expression 3.3-fold and 2.5-fold, respectively (Figure 3E), and I3M increased TRAIL expression in a dose-dependent manner (Figure 3F). This study also investigated whether DR5 suppression by a specific anti-DR5 chimera antibody abrogates I3M-induced apoptosis, and as shown in Figure 3G, DR5-specific chimera antibody treatment significantly reduced the I3M-mediated early and late apoptotic population in HCT116 p53^+/+^ cells. Collectively, these results indicate that I3M enhances apoptosis in HCT116 p53^+/+^ cells by increasing p53-mediated DR5 and TRAIL expression.

### 3.4. I3M-Induced DR5 Upregulation Is Mediated via CHOP Induction

**Figure 4:**
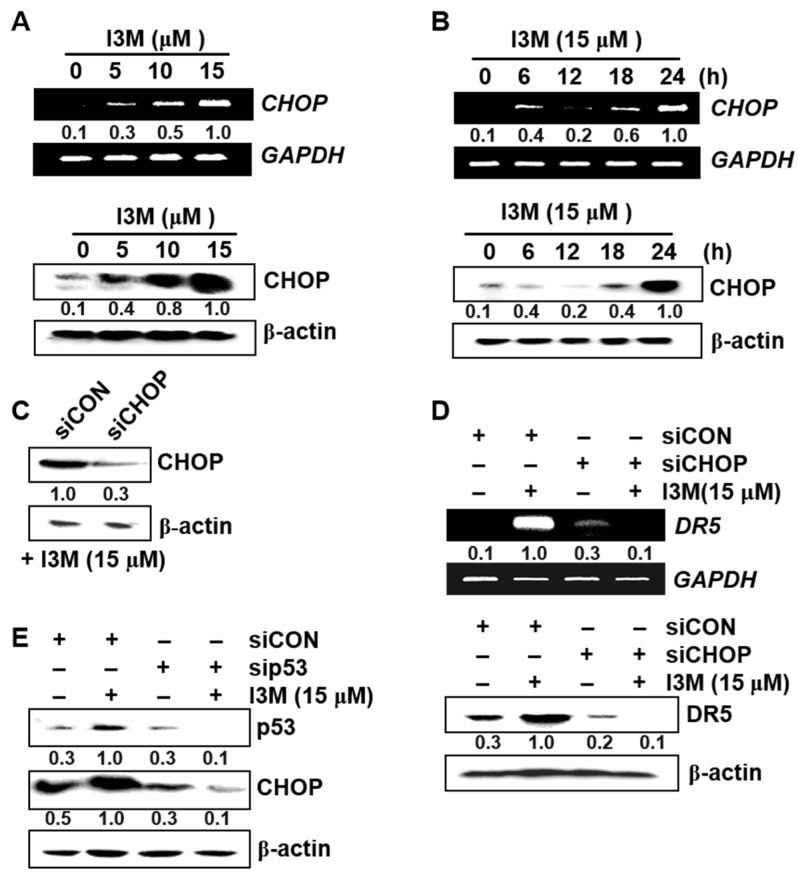
I3M upregulates C/EBP homologous protein (CHOP)-mediated DR5 expression in HCT116 p53^+/+^ cells by inducing p53 expression. (**A**,**B**) HCT116 p53^+/+^ cells were seeded at 1 × 10^5^ cells/mL and incubated with the indicated I3M concentrations (0–15 μM) for 24 h and with 15 μM I3M for the indicated time. RT-PCR and western blotting was performed to measure CHOP expression. (**C**–**E**) HCT116 p53^+/+^ cells were transiently transfected with *CHOP (siCHOP)* and *p53* siRNA (sip53) for 48 h. (**C**) The transfection efficiency was assayed using western blotting. After transient CHOP (**D**) and p53 (**E**) siRNA transfection, for both RT-PCR and western blotting, the cells were treated with I3M (15 μM) for 24 h. DR5 expression was detected at 24 (**D**). In a parallel experiment, p53 and CHOP expression were determined using western blotting at 24 h. *GAPDH* and β-actin were used as internal controls for RT-PCR and western blotting, respectively. The results are the average of three independent experiments and representative relative density mean values are indicated.

Since CHOP is involved in endoplasmic reticulum (ER) stress-related apoptosis by enhancing DR5 expression [14], its role in I3M-induced DR5 upregulation was investigated and the results showed that I3M-induced CHOP was significantly upregulated at both the transcriptional and translational levels compared with the untreated control group (Figure 4A, top; mRNA and bottom; protein). In a parallel experiment, I3M (15 μM) treatment enhanced CHOP expression in a time-dependent manner, with highest mRNA and protein levels was observed at both 24 h (Figure 4B, top; mRNA and bottom; protein). Thereafter, to clarify the functional role of CHOP in I3M-induced DR5 upregulation, transient *CHOP* knockdown using siRNA was performed, which significantly decreased CHOP expression (Figure 4C). The results showed that I3M-mediated DR5 expression was significantly abrogated by siCHOP transfection at both the transcriptional (Figure 4D, top) and translational (Figure 4D, bottom) levels, suggesting that I3M-induced DR5 upregulation is mediated by inducing CHOP expression in HCT116 p53^+/+^ cells. Compared with siCON transfection, sip53 transfection significantly suppressed p53 and CHOP expression (Figure 4E). These data indicate that I3M stimulates CHOP upregulation in HCT116 p53^+/+^ cells by activating p53, which increases DR5 expression.

### 3.5. I3M-Mediated p53 Increases Oxidative Stress-Mediated Apoptosis

**Figure 5:**
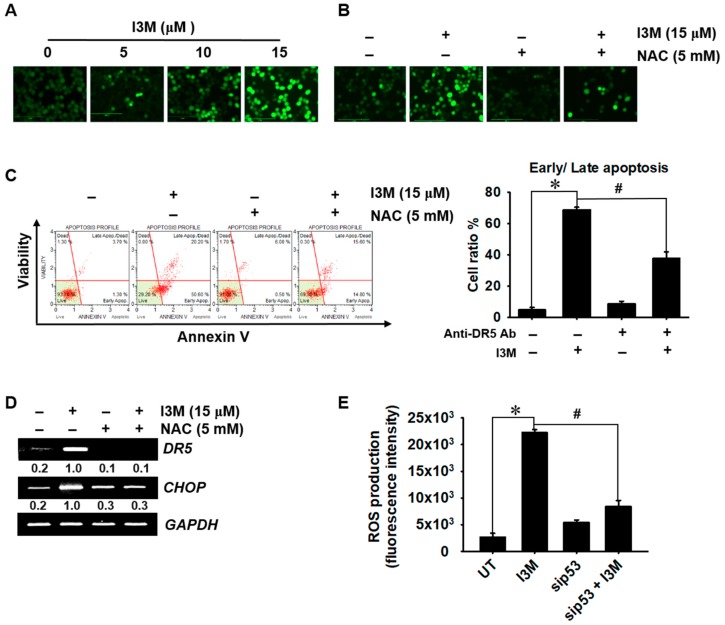
I3M-mediated p53 upregulation increases oxidative stress-mediated apoptosis. (**A**) HCT116 p53^+/+^ cells were treated with the indicated concentrations (0–15 μM) of I3M for 24 h and then stained with 10 µM 2′7′-dichlorofluorescin diacetate (DCFDA). Live imaging was performed with CELENA^®^ S digital imaging system to detect the intracellular ROS levels. (**B**) HCT116 p53^+/+^ cells were pretreated with NAC (5 mM) for 2 h. Intracellular ROS levels were detected by live cell imaging. (**C**) Under NAC-pretreated conditions, apoptotic cell population was measured using MUSE^®^ flow cytometer. (**D**) DR5 and CHOP (**E**) expression were detected using RT-PCR at 24 h. *GAPDH* was used as a loading control. (**E**) HCT116 p53^+/+^ cells were transiently transfected with *p53* siRNA (sip53) for 48 h and then treated with 15 μM I3M for 24 h. H_2_O_2_ generation was analyzed using a fluorometer. The results are the average of three independent experiments and are presented as the mean ± SEM. ^#^, *p* < 0.001 vs. the untreated group (UT); *, *p* < 0.001 vs. the I3M-treated group.

This study also investigated whether I3M-mediated p53 upregulates ROS production. Fluorescence microscopic data showed that I3M increased the intracellular ROS levels in a dose dependent manner (Figure 5A). Pretreatment with *N*-acetylcysteine (NAC) remarkably downregulated the I3M-induced ROS production (Figure 5B). Additionally, we investigated whether ROS generation was associated with I3M-induced apoptosis. Pretreatment with NAC remarkably downregulated I3M-induced annexin V^+^ apoptotic cell population (Figure 5C). Further, RT-PCR data showed that I3M-induced increased levels of DR5 and CHOP were significantly lowered in the presence of NAC (Figure 5C), indicating that ROS played a pivotal role in CHOP-mediated DR5 expression. Furthermore, sip53 transfection partially downregulated I3M-induced ROS production (Figure 5D), suggesting that I3M-mediated p53 plays a key role in ROS-mediated apoptosis in HCT116 p53^+/+^ cells and in *DR5* and *CHOP* expression. Collectively, these results showed that I3M stimulates p53, which promotes ROS-mediated apoptosis by activating CHOP-induced DR5 expression.

### 3.6. Co-Treatment with I3M and TRAIL Potentiates Apoptosis in HCT116, HT 29, and SW480 Colon Cancer Cells

**Figure 6:**
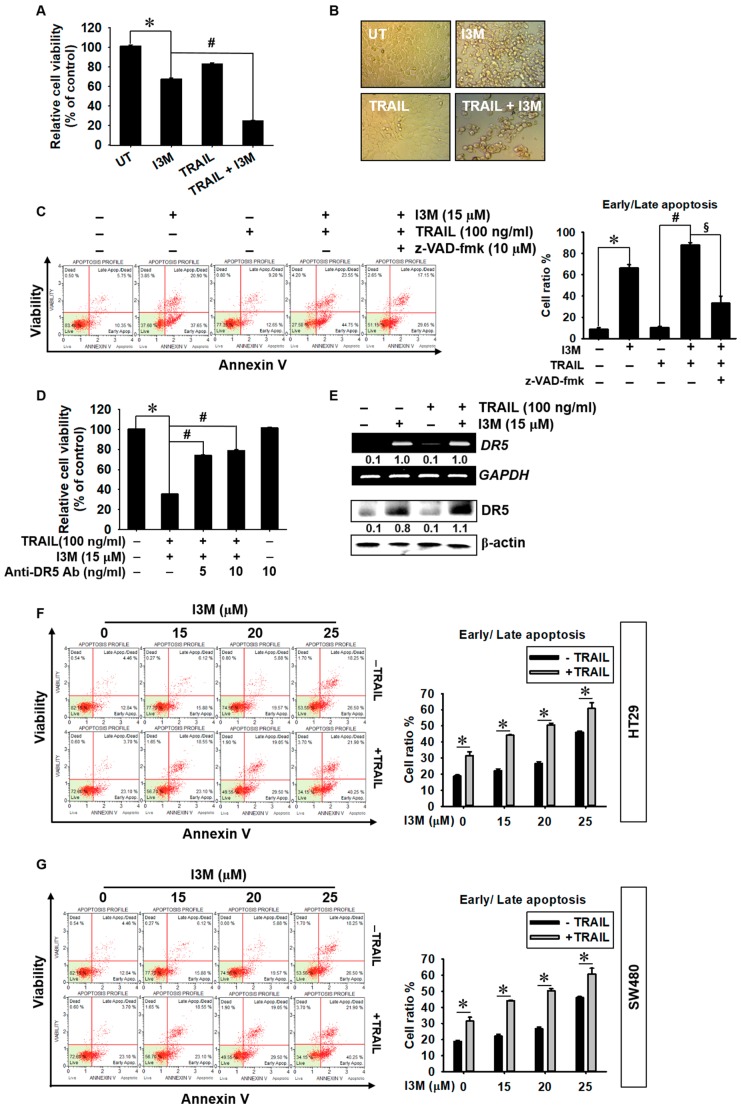
TRAIL enhances I3M-mediated apoptosis in HCT116 p53^+/+^, HT29, and SW480 colon cancer cells. (**A**,**B**) HCT116 p53^+/+^ cells were seeded at a density of 1 × 10^5^ cells/mL and incubated with 15 μM I3M 2 h before treatment with 100 ng/mL TRAIL. (**A**) Cell viability was measured using the MTT assay. (**B**) Cell morphology was examined under a light microscope. (**C**) In a parallel experiment, the cells were incubated with I3M and TRAIL, followed by 10 μM z-VAD-fmk treatment for 2 h. Apoptotic annexin V^+^ population was analyzed using flow cytometry. (**D**) HCT116 p53^+/+^ cells were incubated with I3M (15 μM) and TRAIL for 24 h in the presence or absence of a DR5-specific blocking chimera antibody. Cell viability was measured using the MTT assay. (**E**) After co-treatment with I3M and TRAIL, RT-PCR and western blotting were performed to determine DR5 expression. (**F**) HT26 and (**G**) SW480 colon cancer cells were preincubated with the indicated concentrations of I3M 2 h before 100 ng/mL TRAIL and apoptotic annexin V^+^ population was analyzed using flow cytometry. The results are the average of three independent experiments and are presented as mean ± SEM. *, *p* < 0.001 vs. the untreated control group (UT); ^#^ and ^§^, *p* < 0.001 vs. the I3M and TRAIL co-treated group (**C**). *, *p* < 0.001 vs. the I3M only treated group (**F**,**G**).

Although TRAIL specifically enhances cancer cell apoptosis, many cancer cells have acquired TRAIL-resistance, which hinders its clinical use [15]. Therefore, TRAIL-mediated apoptosis promoting pharmacological agents have been the focus of TRAIL-mediated anti-cancer therapy sensitizers. In this study, we investigated whether recombinant TRAIL boosts I3M-mediated apoptosis via DR5 in HCT116 cells. Interestingly, co-treatment with I3M and TRAIL significantly decreased relative cell viability (Figure 6A), accompanied by increased cellular rounding and detaching (Figure 6B), compared with the I3M only treated group. Flow cytometry data also showed that co-treatment with I3M and TRAIL increased annexin V^+^ populations to as much as 25% higher than that of the I3M only-treated group, which is inhibited by the presence of a pan-caspase inhibitor, z-VAD-fmk, indicating that co-treatment with I3M and TRAIL induced apoptosis via caspase activation (Figure 6C). To confirm the functional role of I3M-induced DR5 in the sensitization of TRAIL-induced apoptosis, the effect of a DR5-specific blocking chimera antibody on co-treatment-induced apoptosis was investigated. As expected, the anti-DR5 chimera antibody significantly attenuated the co-treatment-induced cell death in a dose-dependent manner (75% and 80% cell viability at 5 and 10 ng/mL anti-DR5 Ab, respectively; Figure 6D); meanwhile, DR5 expression was sustained (Figure 6E). The effect of I3M on TRAIL-mediated apoptosis was further tested in two additional colon cancer cells such as HT29 and SW480. Both the cell lines were not associated with apoptosis at 20 µM I3M; however, 25 µM I3M induced the apoptosis approximately up to 40% in each (Figure 6F,G). We noticed that HT26 and SW480 were resistant to TRAIL-mediated apoptosis. Interestingly, all concentrations of I3M tested in this study significantly induced apoptosis under TRAIL-sensitizing conditions in both HT29 and SW480 colon cancer cells. These results suggest that I3M enhances TRAIL-mediated apoptosis in colon cancer cells.

## 4. Discussion

**Figure 7:**
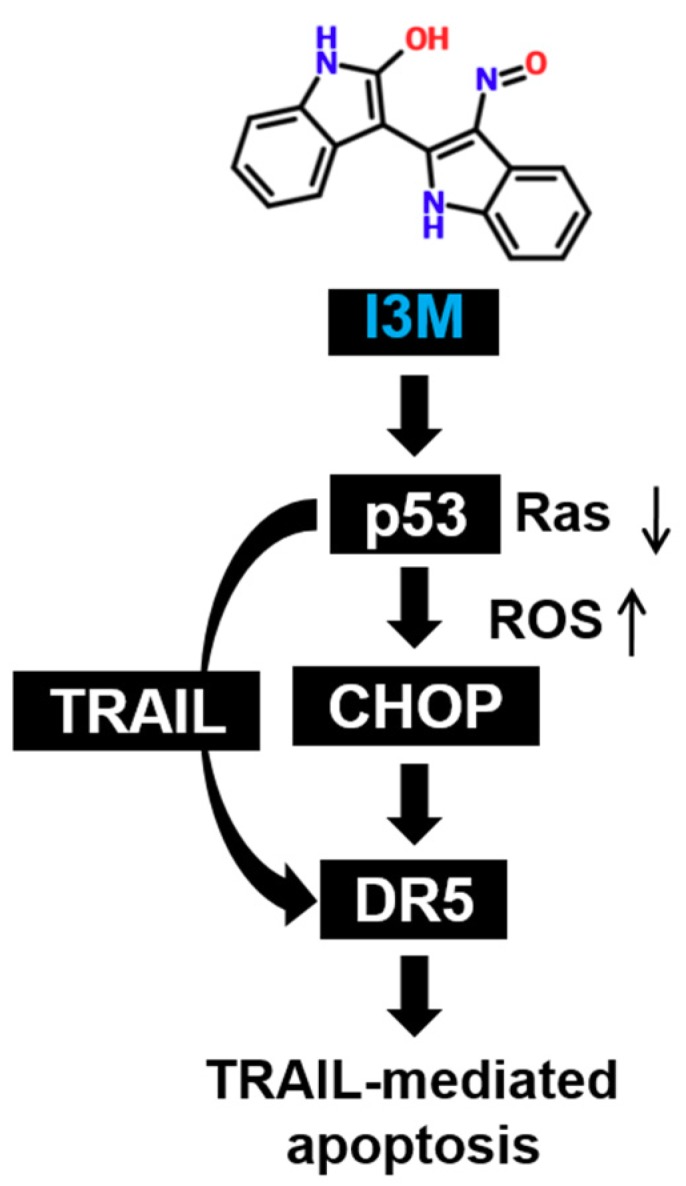
Schematic representation of I3M-induced apoptosis in HCT116 colon cancer cells. I3M is regarded as a cell permeable anti-cancer agent. It downregulates Ras activation and ultimately increases p53 levels in HCT116 p53^+/+^ cells. Increased p53 levels are associated with substantial ROS production in HCT116 p53^+/+^ cells, which ultimately stimulates CHOP-mediated DR5 expression. Additionally, I3M increases the expression of TRAIL, which ligates with its specific receptor, DR5, initiating the apoptotic pathway.

In recent study, I3M showed neuronal protective effects against β-amyloid (Aβ)-mediated apoptosis by inhibiting GSK-3β, which could be applied in the treatment of neurodegenerative diseases such as Alzheimer’s disease [16], because it directly inhibits important neuronal disease factors, including tau, α-synuclein, and Aβ [17]. Additionally, targeting GSK-3β is considered a promising anti-cancer therapeutic strategy by inhibiting NF-κB-targeted gene expression [18]. Previous studies have also demonstrated that I3M, which has the potential to arrest tumor growth in vitro and in vivo, is a promising anti-cancer agent, based on its capability to selectively induce apoptotic cell death in a wide spectrum of human cancer cells with minimal toxicity to normal cells [8,9,10]. However, the molecular mechanisms underlying I3M-induced apoptosis in cancer cells remain incompletely elucidated. In this study, convincing evidence that I3M induces apoptosis via TRAIL sensitization, by activating CHOP-mediated DR5 expression, which is activated by p53-induced ROS generation, is provided (Figure 7).

TRAIL, a member of the tumor necrosis factor (TNF) family of ligands, is capable of initiating apoptosis by ligating with death receptor [19]. It selectively induces apoptosis in a variety of tumor cells and transformed cells, but not normal cells. Consequently, it has received interest as a promising agent in anti-cancer therapy [20]. Particularly, the tumor suppressor p53, is well known to positively modulate TRAIL expression by binding to the promoter region of TRAIL at 346 and 625 bp upstream of the transcription start site, suggesting that it triggers TRAIL-mediated cancer cell death [21]. Recently, Willms et al., also reported that because p53 can activate apoptotic proteins such as Bax and Bid, it is an important inducer of DR-mediated apoptosis in cancer cells [22]. This study revealed that I3M enhanced apoptosis in HCT116 p53^+/+^ cells by activating DR5 and TRAIL expression. However, HCT116 p53^−/−^ cells were less sensitive to I3M, indicating that I3M-mediated apoptosis is triggered in the presence of p53. Additionally, I3M significantly downregulated oncogenic Ras expression in HCT116 p53^+/+^ cells. Ras is known as an upstream p53 molecule and activated Ras stimulates Mdm2 to degrade p53 [12]. However, *p53* gene mutations expression are reportedly associated with upregulated Ras levels [23], indicating an interplay between p53 and Ras in cancer cells. The results of the present study show that p53 activation and Ras loss enhanced I3M-mediated apoptosis in HCT116 p53^+/+^ cells. However, Ras levels were significantly increased in HCT116 p53^−/−^ cells; thus, sustaining cancer cell survival. However, whether I3M directly regulates p53 activation and Ras loss needs to be evaluated further in detail, because this study did not demonstrate direct interplay between p53 and Ras.

p53 is known as a redox-regulating transcription factor that maintains cellular redox status by activating antioxidant gene expression [5]. However, its hyperactivation stimulates pro-oxidant genes, causing ROS imbalance, which consequently induces ROS-mediated apoptosis. Additionally, many previous studies have demonstrated ROS- and p53-mediated DR upregulation [24,25], which triggers TRAIL-mediated apoptosis. The results of the present study showed that transient *p53* knockdown attenuated I3M-induced ROS production in HCT116 p53^+/+^ cells and that NAC treatment downregulated DR5 expression and cancer cell death, indicating that I3M-mediated p53 enhances ROS production and consequently triggers TRAIL-mediated apoptosis by inducing DR5 expression. Additionally, transient *p53* knockdown completely inhibited I3M-mediated apoptosis and inhibited ROS production. Particularly, the possibility that I3M induces apoptosis via GSK-3β and CDK inhibition cannot be excluded because I3M is a known GSK-3β and CDK inhibitor [11]. Kotliarova et al., demonstrated that GSK-3β inhibition stimulated DR expression by inducing c-Myc activation, resulting in apoptosis [26]. Therefore, further experiments to evaluate the relationship between p53 and GSK-3β in I3M-mediated DR5 and TRAIL expression, which result in apoptosis, are needed.

In summary, this study showed that by upregulating DR5 expression, I3M potently enhanced p53-mediated ROS production, thereby triggering TRAIL-mediated apoptosis. We suggest that I3M is a promising anticancer candidate for p53-mediated apoptosis stimulation for overcoming TRAIL-resistant cancers.

## 5. Conclusions

This study revealed that I3M significantly stimulated apoptosis in HCT116 p53^+/+^ colon cancer cells, but not in HCT116 p53^-/-^ cells, indicating that I3M-mediated apoptosis absolutely depends on p53 status. Particularly, by activating ROS-induced CHOP activation, I3M-mediated p53 triggered DR5 and TRAIL expression. In addition, I3M sensitized TRAIL-resistant HT29 and SW480 colon cancer cells to TRAIL-mediated apoptosis. Thus, it can be concluded that by its ability to overcome low p53 levels, I3M and TRAIL co-treatment is a promising anti-cancer therapy. However, further studies to investigate whether I3M directly regulates p53 status via GSK-3β and CDK inhibition, are warranted.
